# COG6‐CDG: Novel variants and novel malformation

**DOI:** 10.1002/bdr2.1981

**Published:** 2022-01-23

**Authors:** Lara Cirnigliaro, Paolo Bianchi, Luisa Sturiale, Domenico Garozzo, Giovanna Mangili, Liesbeth Keldermans, Renata Rizzo, Gert Matthijs, Agata Fiumara, Jaak Jaeken, Rita Barone

**Affiliations:** ^1^ Child Neurology and Psychiatry Section, Department of Clinical and Experimental Medicine University of Catania Catania; ^2^ Neonatology Unit Giovanni XXIII Hospital Bergamo Italy; ^3^ CNR, Institute for Polymers, Composites and Biomaterials IPCB Catania; ^4^ Department of Human Genetics KU Leuven Leuven Belgium; ^5^ Referral Centre for Inherited Metabolic Disease Department of Clinical and Experimental Medicine, University of Catania Catania; ^6^ Department of Development and Regeneration, Centre for Metabolic Diseases University Hospital Gasthuisberg, KU Leuven Catania Belgium

**Keywords:** *COG6*, congenital disorder of glycosylation (CDG), corpus callosum dysgenesis, combined N‐ and O‐glycosylation defect, congenital ano‐rectal malformations

## Abstract

**Background:**

Deficiency of Conserved Oligomeric Golgi (COG) subunits (COG1–8) is characterized by both N‐ and O‐protein glycosylation defects associated with destabilization and mislocalization of Golgi glycosylation machinery components (COG‐CDG). Patients with COG defects present with neurological and multisystem involvement and possible malformation occurrence. Eighteen patients with COG6‐CDG (*COG6* mutations) were reported to date. We describe a patient with COG6‐CDG with novel variants and a novel clinical feature namely a congenital recto‐vaginal fistula.

**Methods:**

In‐depth serum N‐ and O‐glycosylation structural analyses were conducted by MALDI‐TOF mass spectrometry. *COG6* variants were identified by a gene panel and confirmed by Sanger sequencing.

**Results:**

This female newborn presented with facial dysmorphism, distal arthrogryposis and recurrent stool discharges per vaginam. A double‐contrast barium‐enema X‐ray study revealed a dehiscence (approximately 5 mm) at the anterior wall of the rectal ampoule communicating with the vagina consistent with a recto‐vaginal fistula. She had developmental delay, corpus callosum dysgenesis, liver and gastrointestinal involvement, hyperthermia episodes and early demise. Serum N‐ and O‐glycosylation analyses pointed to a profound Golgi disarrangement. We identified two novel variants in *COG6:* a deletion of 1 bp mutation c.823delA creating a shift in the reading frame and a premature stop codon and a 3 bp deletion (c.1141_1143delCTC) producing an in‐frame deletion of 1 amino acid.

**Conclusion:**

The congenital recto‐vaginal fistula is a rare type of anorectal malformation that, to our knowledge, has not been reported in patients with a COG6 defect nor in patients with other COG defects. This study broadens COG6‐CDG genetic landscape and spectrum of malformations.

## 1 INTRODUCTION

Congenital disorders of glycosylation (CDG) are a group of genetic diseases caused by defects in the synthesis and attachment of the oligosaccharide moieties (glycans) of glycoproteins and glycolipids. Most CDG affect protein glycosylation, which consists in the covalent linkage of glycan chains to proteins at their glycosylation sites formed by asparagine (N‐glycosylation) or serine/threonine residues (O‐glycosylation), respectively (Francisco et al., 2019).

Disorders of N‐glycosylation can be subdivided into CDG‐I (defects in the assembly of N‐glycan’s oligosaccharide precursor in the cytosol and the ER) and CDG‐II (abnormal N‐glycan processing mostly at the level of the Golgi apparatus). Serum transferrin glycoform analysis is the first‐line laboratory test for the diagnosis of N‐glycosylation defects: an increase of asialylated and/or disialylated transferrin or increased mono‐, di‐ tri‐ and/or asialotransferrin are observed in CDG‐I and CDG‐II, respectively. The analysis of mucin type O‐glycosylated apolipoprotein CIII (apoCIII) is informative for the diagnosis of protein O‐glycosylation as well as combined N‐ and O‐glycosylation disorders (Linders, Peters, Ter Beest, Lefeber, & van den Bogaart, 2020).

A subgroup of CDG type II comprises defects in the Conserved Oligomeric Golgi complex, composed of eight subunits (COG1–8). These subunits are organized into two distinct lobes: lobe A consisting of COG1–4 and lobe B consisting of COG5–8. The COG complex contributes to membrane trafficking of proteins within the Golgi and to retrograde transport from the Golgi to the ER. A major phenotype of COG dysfunction is the occurrence of both N‐ and O‐glycosylation defects associated with destabilization and mislocalization of Golgi glycosylation machinery components. Detrimental variants in each of the COG subunits have been associated with CDG, except for COG3 (Reynders, Foulquier, Annaert, & Matthijs, 2011). COG‐CDG clinical presentation includes developmental disability, hypotonia, microcephaly, dysmorphic features, skin abnormalities, short stature, skeletal anomalies, liver and gastrointestinal dysfunction as well as congenital malformations.

Eighteen patients with COG6‐CDG have been reported (Alsubhi et al., 2017; Althonaian, Alsultan, Morava, & Alfadhel, 2018; Huybrechts et al., 2012; Komlosi et al., 2020; Li et al., 2019; Lubbehusen et al., 2010; Lugli et al., 2021; Mandel et al., 2020; Rymen et al., 2015; Zhao et al., 2021). Here we describe a COG6‐CDG patient with novel variants presenting with dysmorphism, severe neurological disability, corpus callosum dysgenesis, and a novel clinical feature namely a congenital recto‐vaginal fistula. We review the clinical spectrum of COG6‐CDG with emphasis on malformations.

## 2 METHODS

### 2.1 Ethics considerations

This study was based solely on information and investigations that were carried out as part of the routine clinical care of CDG patients. All procedures performed in studies involving human participants were in accordance with the ethical standards of the institutional research committee at Policlinico “G. Rodolico‐San Marco” Catania and with the 1964 Helsinki declaration and its later amendments. Written informed consent was signed by the parents of the proband.

### 2.2 Serum glycosylation analyses

Serum transferrin isoforms were quantified through capillary zone electrophoresis (CZE) as reported (Carchon, Chevigné, Falmagne, & Jaeken, 2004). In‐depth total serum N‐glycan structural analyses were conducted by matrix‐assisted laser desorption/ionization time‐of‐flight (MALDI‐TOF) mass spectrometry (MS). MS analyses of permethylated serum N‐glycans and of apoCIII O‐glycosylation were carried out as described (Palmigiano et al., 2017).

### 2.3 Next generation sequencing

The analysis was performed by a gene panel using massive parallel sequencing. Samples were prepared from genomic DNA with the Illumina TruSeq DNA sample preparation kit and enriched for 79 glycosylation‐related genes using a custom in‐solution targeted assay (NimbleGen SeqCap EZ kit; Roche). The enriched libraries were paired end sequenced on HiSeq2500 (Illumina). The resulting reads were mapped to the reference genome and variants were detected with GATK Haplotype Caller after duplicate removal, realignment around indels, and base quality score recalibration. Compound heterozygous variants found in *COG6* (NM_001145079) were confirmed by Sanger sequencing.

## 3 RESULTS

### 3.1 Clinical presentation

**FIGURE 1:**
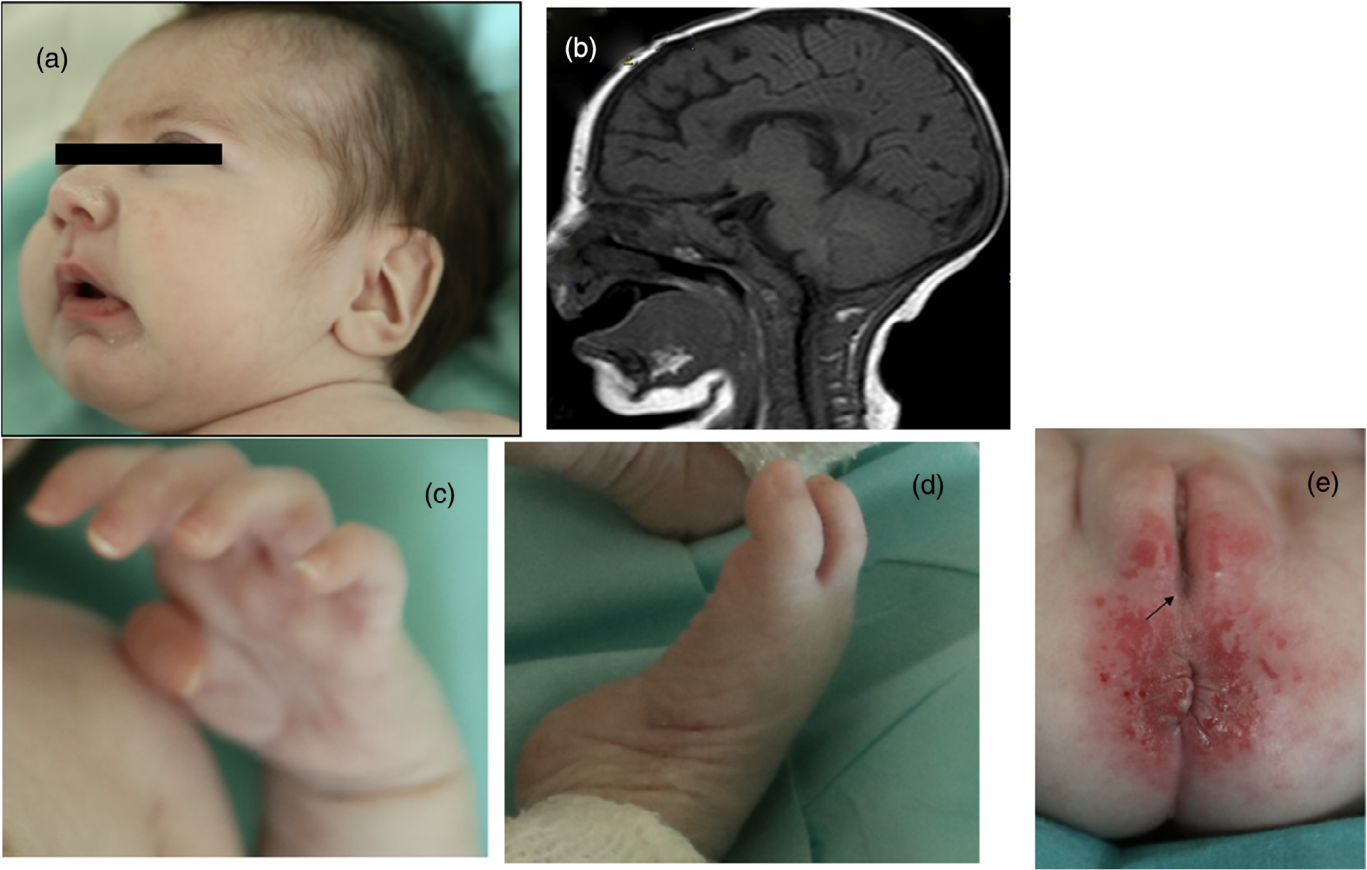
Patient with COG6‐CDG at the age of 3 months. (a) Facial dysmorphism (sloping forehead, thick nasal alae, mild retrognathia, large ears). (b) T1‐weighted sagittal brain magnetic resonance imaging shows partial agenesis of the corpus callosum and normal cerebellum. (c) distal arthrogryposis: opposed thumb, clenched fingers and (d) talipes equinovarus. (e) Normal anus and external genitalia, fecal discharges (not shown) per vaginam (arrow) were caused by congenital recto‐vaginal fistula

The baby was a female, first‐ born to healthy, unrelated Italian parents. Weight at birth (37th week) was 2080 g, length 42 cm and head circumference 31 cm (all below the third percentile). Abnormal clinical features included facial dysmorphism (epicanthal fold, sloping forehead, small and pinched nose, large and dysplastic ears), opposed thumbs, clenched and slender fingers, and right talipes equinovarus. Moreover, recurrent stool discharges per vaginam revealed a recto‐vaginal fistula with normal external genitalia. No other anorectal malformation was noted (Figure 1).

At the age of 7 days, she was hospitalized because of profuse diarrhea and weight loss of more than 10%, despite adequate water and caloric intake. Neurological examination revealed poor spontaneous movements, muscular hypotrophy and generalized hypotonia.

She suffered from recurrent episodes of hyperthermia without increased inflammation indices (normal C‐reactive protein). Serum immunoglobulins levels and lymphocyte population analyses were normal.

At the age of 3 months parenteral nutrition was started because of periodical episodes of severe failure to thrive associated with diarrhea and abdominal distension. Biochemical investigations revealed conjugated hyperbilirubinemia (total bilirubin up to 3.1 mg/dl; conjugated bilirubin 1.8 mg/dl), increased liver enzymes (AST up to 774 U/L; ALT 293 U/L; LDH 2398 U/L). Coagulation tests showed normal prothrombin time and decreased protein C (40.6%, normal range: 69–134). Serum transferrin electrophoresis revealed a type 2 pattern (elevated 3‐sialotransferrin (27.5%; normal range: 3.6–5.5) and, to a lesser extent, 2‐sialotransferrin (7%; normal range: 0–1.1). Genetic studies showed a normal female karyotype and normal CGH array analysis.

Brain magnetic resonance imaging (MRI) showed enlarged lateral ventricles and hypoplasia of the genu and rostral part of the corpus callosum with a normal cerebellum (Figure 1). Electroencephalogram, electrocardiogram, echocardiography, and abdominal ultrasonography were normal.

A double‐contrast barium‐enema X‐ray study revealed a dehiscence (approximately 5 mm) at the anterior wall of the rectal ampoule communicating with the vagina consistent with the presence of a recto‐vaginal fistula.

The clinical course was characterized by failure to thrive, recurrent infections and hyperthermia episodes. The child developed severe psychomotor disability: at the age of 14 months she was unable to hold her head up. She passed away at the age of 20 months during a febrile episode owing to a respiratory infection with lung failure.

## 4 MUTATION ANALYSIS

Next‐generation CDG panel analysis revealed two heterozygous mutations: p.Ser275Valfs*31 (c.823delA) in exon 9 and p.Leu381del (c.1141_1143delCTC) in exon 12 in the COG6 gene (NM_020751.3), inherited from her father and her mother, respectively.

The deletion of 1 bp mutation p.Ser275Valfs*31 (c.823delA) creates a frameshift which will lead to a premature stop codon. This mutation is listed in gnomAD database v2.1 with an allele frequency of 0.00001193, not present in the homozygous state. This mutation is a frameshift in a gene for which loss‐of‐function is a known mechanism of disease. According to the standards and guidelines recommended by the American College of Medical Genetics and Genomics (ACMG) (Richards et al., 2015), this mutation is classified as pathogenic.

The 3 bp deletion p.Leu381del (c.1141_1143delCTC) creates an in‐frame deletion of one amino acid. This mutation is listed in gnomAD database v2.1 with an allele frequency of 0.00001208, not present in the homozygous state. We have seen this mutation before in the homozygous state in another CDG type II patient with similar phenotype. Based on this data, and according to the ACMG guidelines, this mutation is classified as likely pathogenic.

### 4.1 Serum glycosylation analyses by MALDI mass spectrometry (MALDI‐MS)

**FIGURE 2:**
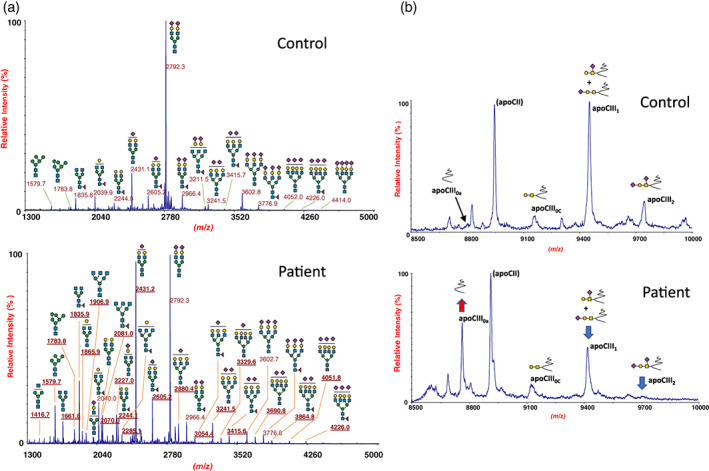
MALDI‐TOF analysis of N‐glycosylation (a) and O‐glycosylation (b) in the patient with COG6‐CDG at age 6 months. Each MALDI‐TOF profile reports the relative intensity (%) of the individual structures with respect to the most abundant molecular ion (base peak). (a) Compared to a pediatric control, the patient shows a severely altered serum N‐glycan profile with increased amounts of unprocessed or impaired processed structures such as oligomannose and truncated complex glycans lacking sialic acid, galactose and/or N‐acetylglucosamine, evidenced by underlined *m/z* values. Increased levels of all the fucosylated structures were also observed. (b) MALDI‐TOF analysis of serum apoCIII glycoprotein shows an increase of aglycosylated isoform (apoCIII_0_) and decrease of mono‐ and di‐glycosylated isoforms (apoCIII_1_ and apoCIII_2_) consistent with a mucin‐type core‐1 O‐glycosylation defect. Symbols: blue square, N‐acetylglucosamine; green circle, mannose; yellow circle, galactose; purple diamonds, sialic acid; red triangle, fucose; yellow square, N‐acetylgalactosamine

MALDI‐MS profile of serum N‐glycans showed remarkable alterations such as severe hyposialylation and hypogalactosylation together with the occurrence of abnormal structures lacking antennary N‐acetylglucosamine. Moreover, significantly increased levels were observed for oligomannose structures and for all the fucosylated species (Figure 2a). Serum apoCIII MALDI‐MS analyses is widely used for the determination of three apoCIII isoforms (apoCIII_0–2_), either with two sialic acids (apoCIII_2_), with one sialic acid linked to galactose or N‐acetylgalactosamine (apoCIII_1_), or with no sialic acid residue (apoCIII_0_). In the present patient, MS analyses of serum apoCIII showed a dramatic increase of aglycosylated apoCIII_0_, decrease of apoCIII_1_ and nearly absent apoCIII_2_ isoform, consistent with a severe mucin‐type core‐1 O‐glycosylation defect (Figure 2b). Altogether, these analyses showed a combined N‐ and O‐glycosylation defect consistent with impaired Golgi glycosylation.

## 5 DISCUSSION

COG6‐CDG is a multisystem disorder characterized by a broad clinical and genetic spectrum with 19 reported patients, including the present one (Table 1). They had severe neurological and multi‐organ involvement with early lethality in a considerable number of patients mostly harboring detrimental *COG6* variants. Thirteen patients (68%) died before 2 years of age owing to internal organ failure, coagulopathy, or brain oedema. Most patients had severe developmental disability, microcephaly, brain anomalies and facial dysmorphism. Skeletal involvement including arthrogryposis multiplex, kyphoscoliosis, and osteopenia was frequently reported as well as skin abnormalities such as hyperkeratosis, dry skin, ichthyosis, and ectodermal signs with hypohidrosis/hyperthermia. Transferrin glycosylation analyses showed a type 2 pattern in agreement with defective Golgi trafficking in COG6‐CDG (Lubbehusen et al., 2010; Rymen et al., 2015) except in Shaheen syndrome. The latter patients have mild to moderate intellectual disability, facial dysmorphism, mild ectodermal dysplasia and a normal serum sialotransferrin pattern associated with the deep intronic splice site mutation c.1167‐24A>G (Shaheen et al., 2013).

**TABLE 1:** Demographic, clinical and molecular features of patients with COG6‐CDG

Patient reference	P1 [15]	P2 [9]	P3 [26]	P4 [26]	P5 [26]	P6 [26]	P7 [26]	P8 [26]	P9 [26]
Gender	F	F	F	M	F	M	M	F	F
Country	Turkey	Morocco	Bulgaria	Turkey	Turkey	Morocco	Morocco	Morocco	Turkey
Genotype	Hom c.G1646T	Hom c.G1646T	Hom c.511C>T	c.1746 + 2 T>G (splicing defect)	c.1238_ 1239insA	c.1646G>T c.785A>G	c.1646G>T c.785A>G	Hom c.1646G>T	c.511C>T c.1746 + 2G>T (splicing defect)
Protein change	G549V	G549V	R171*	−	F414Lfs*4	G594V Y262C	G594V Y262C	G594V	R171*
Deceased (cause)	5 wks (brain edema)	6 yrs (nr)	4 wks (lung failure)	12 mos (nr)	15 mos (liver failure)	Alive 21 yrs	14 mos (liver transplant)	5 wks (DIC)	Alive 12 yrs
Neurological features									
DD/ID	nr	+	+	+	+	+	+	nr	+
Microcephaly	nr	+	+	+	+	+	+	+	+
Hypotonia	nr	+	+	+	−	−	−	−	+
Seizures	+	−	+	−	−	−	−	−	−
Brain MRI	nr	−	CCA cortical dysplasia enlarged ventricles	Cerebral atrophy	Cerebral and cerebellar atrophy	nr	nr	−	Cortical atrophy
Systemic features									
Facial dysmorphism	nr	Broad palpebral fissures, retrognathia	+	Long philtrum, flat nasal bridge	Broad palpebral fissures, retrognathia	Wide mouth, thin lips, prominent nose	−	nr	Low set frontal hairline, bilateral epicanthic folds, tubular nose, large mouth
Skeleton	nr	Post‐axial polydactyly	Opposed thumbs, club feet, hip dysplasia	nr	Post‐axial polydactyly	−	−	nr	Scoliosis, joint hypermobility
Skin	nr	nr	nr	nr	Hyperkeratosis, inverted nipples	Hyperkeratosis	Dry skin	nr	Orange peel skin, inverted nipples
Hyperthermia Hypohidrosis	nr	nr	+	nr	−	+	+	+	+
Liver/spleen	Increased S‐AST, cholestasis	Increased S‐AST, ALT	Hepato‐splenomegaly, cholestasis	Splenomegaly	Hepato‐splenomegaly	Splenomegaly	Hepato‐splenomegaly, cholestasis, liver failure	Hepato‐splenomegaly, cholestasis	Hepato‐splenomegaly, cholestasis, cirrhosis
Gastro‐intestinal tract	nr	Inflammatory bowel disease	−	Chronic diarrhea	Chronic diarrhea, gastrointestinal bleeding	Gastroenteritis	nr	Bowel ischemia	Chronic diarrhea
Coagulopathy	+	−	−	−	−	+	+	+	+
Failure to thrive	nr	+	+	+	+	+	+	nr	−
Recurrent infections	nr	+	−	+	+	+	+	−	+
Immuno‐deficiency	nr	+	−	nr	+	nr	nr	−	nr
Malformations									
Cardiac	nr	nr	ASD, PDA	nr	ASD, PDA	nr	nr	ASD	VSD
Gastro‐intestinal	nr	Anal anteposition	nr	nr	nr	nr	nr	−	−
Urogenital	nr	−	−	nr	nr	−	nr	−	Right renal agenesis

Patient reference	P10 [1]	P11 [1]	P12 [2]	P13 [12]	P14 [11]	P15 [17]	P16 [17]	P17 [16]	P18 [32]	P19 This study
Gender	M	F	F	M	F	Amb (XY)	Amb (XY)	Amb (XY)	F	F
Country	Saudi Arabia	Saudi Arabia	Saudi Arabia	China	Greece	Israel	Israel	Morocco	China	Italy
Genotype	Hom c.1378G>T	Hom c.10759 T>G	c.116724A>G	c.1A>G c.388C>T	Hom c.511C>T	c.518_540 +3del	c.518_540 +3del	Hom c.782 T>A	c.1843C>T c.428G>T	c.823delA c.1141_1143delCTC
Protein change	V460F	−	−	? Q130*	R171*	−	−	L261*	Q615X S143I	S275Vfs*31 L381del
Deceased (cause)	3 mos (nr)	Alive 3 yrs	Alive 5 yrs	Alive 28 mos	4 days (lung failure)	18 days (lung failure)	30 days (bleeding diathesis)	9 mos (nr)	Alive 20 mos	20 mos (fever, lung failure)
Neurological features										
DD/ID	+	+	+	+	nr	nr	nr	+	+	+
Microcephaly	+	+	+	+	−	+	+	+	+	−
Hypotonia	+	+	+	−	+	nr	hypertonia	+	−	+
Seizures	−	−	−	+	−	−	−	+	−	−
Brain MRI	CCA	CCA	Cerebral atrophy, thin CC	−	nr	CCA, reduced white matter enlarged ventricles	Partial CCA	Partial CCA, cortical dysplasia cerebellar atrophy	Enlarged ventricles	Partial CCA enlarged ventricles
Systemic features										
Facial dysmorphism	+	+	Broad palpebral fissures, retrognathia, wide mouth with thin lips, prominent nose	−	Small pinched nose, dysplastic hears, incomplete eye closure	Hypertelorism, retrognathia, low‐set ears	Bulbus, upturned nose, hypertelorism, low‐set ears	High forehead, anteverted nostrils, long philtrum, low set ears	Narrow forehead, hypertelorism, bulbous nose, downslanting palpebral fissures	Sloping forehead, thick nasal alae, mild retrognathia, large ears
Skeleton	Skeletal dysplasia	−	Generalized osteopenia	−	Arthrogryposis talipes equinovarus	Arthrogryposis multiplex	Kyphoscoliosis, right club foot, clenched hands	Arthrogryposis, bilateral clubfeet	Opposed thumbs, camptodactyly	Clenched fingers, right club foot
Skin	Lipodystrophy	Lipodystrophy	−	Red rash	Dry, rigid skin, erosions,	−	Ichthyosis	−	Dry skin	−
Hyperthermia Hypohidrosis	−	−	+	+	−	−	−	+	+	+
Liver/spleen	−	−	Splenomegaly, increased S‐AST, ALT	−	Hepato‐splenomegaly	Direct hyper‐bilirubinemia	Increased S‐AST, hyper‐bilirubinemia	−	Cholestasis, increased S‐AST, ALT	Increased S‐AST, dALT
Gastro‐intestinal tract	nr	nr	nr	−	nr	Vomiting	−	Vomiting	Chronic diarrhea	Profuse diarrhea
Coagulopathy	−	−	+	−	−	−	+	−	−	+
Failure to thrive	+	+	+	+	−	+	−	+	nr	+
Recurrent infections	nr	nr	+	+	−	−	−	+	+	+
Immuno‐deficiency	nr	nr	−	nr	−	−	−	−	nr	−
Malformations										
Cardiac	Dysplastic aortic valve, PDA	−	−	VSD, ASD	ASD	−	VSD	−	ASD	−
Gastro‐intestinal	nr	nr	nr	−	nr	Gastro‐intestinal tract malrotation	nr	−	nr	−
Urogenital	−	−	−	nr	−	Ambiguous genitalia	Ambiguous genitalia	Ambiguous genitalia:	nr	Recto‐vaginal fistula

*Note*: P6 and P7 are brothers and first‐degree cousins of P8; P8 was the sister of the patient P1; P14 and P15 were siblings.

Abbreviations: −, absent; +, present; ASD, atrial septal defect; DD, developmental disability; DIC, disseminated intravascular coagulation; EEG, electroencephalogram; homo, homozygous; ID, intellectual disability; mos, months; MRI, magnetic resonance imaging; nr, not reported; PDA, patent ductus arteriosus; S, serum; VSD, ventricular septal defect; wks, weeks; yrs, years.

In the present patient, the phenotype and the occurrence of severe combined N‐and O‐glycosylation defects were all consistent with COG6‐CDG. In particular, mass spectrometry analyses of serum total N‐glycans showed an increase of multiple abnormal structures corresponding to heavily truncated N‐glycans lacking terminal sialic acids, galactose and GlcNac residues, as well as an increase of oligomannose N‐glycans. Furthermore a general fucosylation increase was observed. ApoCIII analyses showed an increase of under‐ and unglycosylated structures consistent with a disorder of mucin type O‐glycosylation.

The most severe COG6‐CDG phenotype with early lethality is more frequently associated with loss‐of‐function variants (Lugli et al., 2021; Mandel et al., 2020; Rymen et al., 2015). Variants linked to residual COG6 activity are likely to cause milder phenotypes (Alsubhi et al., 2017; Rymen et al., 2015), as in the case of the deep intronic splice site variant reported in a small subgroup of patients with Shaheen syndrome. These have a better outcome and longer life expectancy (Alsubhi et al., 2017; Althonaian et al., 2018; Shaheen et al., 2013). However, a different clinical course was described in two patients homozygous for the same missense variant c.G1646T in *COG6* (Huybrechts et al., 2012; Lubbehusen et al., 2010).

Noteworthy, patients with COG6‐CDG may display structural brain anomalies mostly consisting of corpus callosum dysgenesis besides various malformations affecting the cardiac, urogenital, and gastrointestinal (Table 1). The present patient showed a congenital recto‐vaginal fistula with normal anus, a rare type of anorectal malformation characterized by the abnormal connection of the rectum to the vagina that, to our knowledge, has not been reported in patients with a COG6 defect nor in patients with other COG defects. This and the other reported malformations are summarized in Table 2 and Figure S1. In order of frequency, cardiovascular malformations were described in 64% of studied patients: atrial septal defects in six, ventricular septal defect (VSD) in three and patent ductus arteriosus (PDA) in three patients (Alsubhi et al., 2017; Althonaian et al., 2018; Komlosi et al., 2020; Li et al., 2019; Mandel et al., 2020; Zhao et al., 2021). In this regard, the combination of congenital heart defects, gastrointestinal and liver problems, recurrent infections, hyperkeratosis and hyperthermia was considered pathognomonic of COG6‐CDG (Rymen et al., 2015). Brain malformation detected in 53% of patients with COG6‐CDG comprise corpus callosum hypoplasia or dysgenesis and less frequently cortical dysplasia or cerebellar vermis hypoplasia (Alsubhi et al., 2017; Althonaian et al., 2018; Lugli et al., 2021; Mandel et al., 2020; Rymen et al., 2015; Zhao et al., 2021). Malformations of the urogenital system were reported in five patients (38%) including ambiguous genitalia in three patients (hypertrophic clitoris, no vaginal introit and a Müllerian residue between bladder and rectum in a female; no palpable testes, micropenis with severe hypospadias in two male siblings) (Lugli et al., 2021; Mandel et al., 2020). A relationship between glycoprotein metabolism and sex development has been hypothesized because glycosylation processes are fundamental for the correct gonad migration and genitalia morphogenesis, and the biological activity in the gonads is dependent on glycosylation of gonadotropins and their receptors (Mandel et al., 2020). Congenital anorectal malformation including recto‐vaginal fistulas, are related to defects in embryonic development of the urinary, genital, and anorectal tracts that result from the division of the embryonic cloaca. Sonic hedgehog (Shh), an endoderm‐derived signaling molecule required for normal development of the distal hindgut in mice, is expressed in the cloaca endoderm and has both early and late functions during anorectal and urogenital development. Mice with mutations in Shh signaling pathways recapitulate the whole spectrum of anorectal malformations that are seen in humans (Gredler, Patterson, Seifert, & Cohn, 2020; Mo et al., 2001). Noteworthy, GALNT1‐mediated glycosylation is required for Shh activation in bladder cancer stem cells suggesting that proper glycosylation may play a role in Shh signaling function (Li et al., 2016).

**TABLE 2:** Malformations according to system in COG6‐CDG patients

Malformations	Number of patients^a^	References
Central nervous system	8/15 (53%)	Alsubhi et al., 2017, Althonaian et al. (2018), Lugli et al. (2021), Mandel et al. (2020), and Rymen et al. (2015)
Corpus callosum hypoplasia	5/8 (62%)	
Corpus callosum dysgenesis	3/8 (37%)	
Cortical dysplasia	2/8 (25%)	
Cerebellar vermis hypoplasia	1/8 (12%)	
Hydrocephalus	1/8 (12%)	
Cardiovascular system	9/14 (64%)	Alsubhi et al. (2017), Komlosi et al. (2020), Li et al. (2019), Mandel et al. (2020), Rymen et al. (2015), and Zhao et al. (2021)
ASD	6/9 (66%)	
VSD	3/9 (33%)	
PDA	3/9 (33%)	
Dysplastic aortic valve	1/9 (11%)	
Gastrointestinal system	2/7 (28%)	Huybrechts et al. (2012) and Mandel et al. (2020)
Anal anteposition	1/7 (14%)	
Malrotation of gastrointestinal tract	1/7 (14%)	
Urogenital system	5/13 (38%)	Lugli et al. (2021), Mandel et al. (2020), and Rymen et al. (2015)
Renal agenesis	1/5 (20%)	
Ambiguous genitalia	3/5 (60%)	
Recto‐vaginal fistula	1/5 (20%)	

^a^ Patients with malformation/studied patients.

Abbreviations: ASD, atrial septal defect; PDA, patent ductus arteriosus; VSD, ventricular septal defect.

Most clinical signs of COG6‐CDG are present in other COG‐CDG but some malformations reported in patients with COG6‐CDG have been detected to a lesser extent in other COG defects such as corpus callosum agenesis/hypoplasia (COG1‐4) (Kodera et al., 2015;  Miura, Tay, Aw, Eklund, & Freeze, 2005; Ng et al., 2011; Salazar et al., 2021) and COG7‐8 (Arora et al., 2019; Medrano et al., 2019; Morava et al., 2007) and malformations of the cardiovascular system (COG1) (Zeevaert et al., 2009) and COG7 (Spaapen et al., 2005; Wu et al., 2004). The relative severity of COG6‐CDG is likely due to the observed instability of COG lobe B, as COG6‐CDG patients also have lower protein levels of COG5 and COG7. Moreover, knockout of individual COG subunits results in severe fragmentation and dilation of Golgi cisternae. It also affects the endolysosomal system, delays retrograde protein trafficking, causes protein missorting and changes the secretion profile (Blackburn, D’Souza, & Lupashin, 2019; D’Souza, Taher, & Lupashin, 2020). The intertwined interaction of COG subunits is also supported by the present glycosylation findings showing in COG6‐CDG similar hypogalactosylation and hyposialylation as in other COG defects (Linders et al., 2020; Palmigiano et al., 2017). Further studies are needed to characterize the impact of COG subunit deficiency on the patho‐mechanisms of birth defects observed in COG‐CDG, particularly in COG6‐CDG.

## AUTHOR CONTRIBUTIONS

Rita Barone and Lara Cirnigliaro contributed to conception and design and drafted the manuscript. Paolo Bianchi and Giovanna Mangili were involved in acquisition of clinical data. Luisa Sturiale and Domenico Garozzo were responsible for glycosylation mass spectrometry analyses. Liesebeth Keldermans carried out molecular analyses. Renata Rizzo, Agata Fiumara, Gert Mattijs, Rita Barone and Jaak Jaeken were involved in data interpretation and they all revising the manuscript critically for important intellectual content. All authors approved the final manuscript version submitted.

## Supporting information


**Figure S1** Overview of clinical features reported in patients with COG6‐CDG (N:19)

## Data Availability

The data that support the findings of this study are available from the corresponding author upon reasonable request.
