# Supplementary figures and images for: COG6‐CDG: Novel variants and novel malformation
Source: Birth Defects Res. 2022 Jan 23;114(5-6):165–74. doi: 10.1002/bdr2.1981 (PMC9306771; doi:10.1002/bdr2.1981)

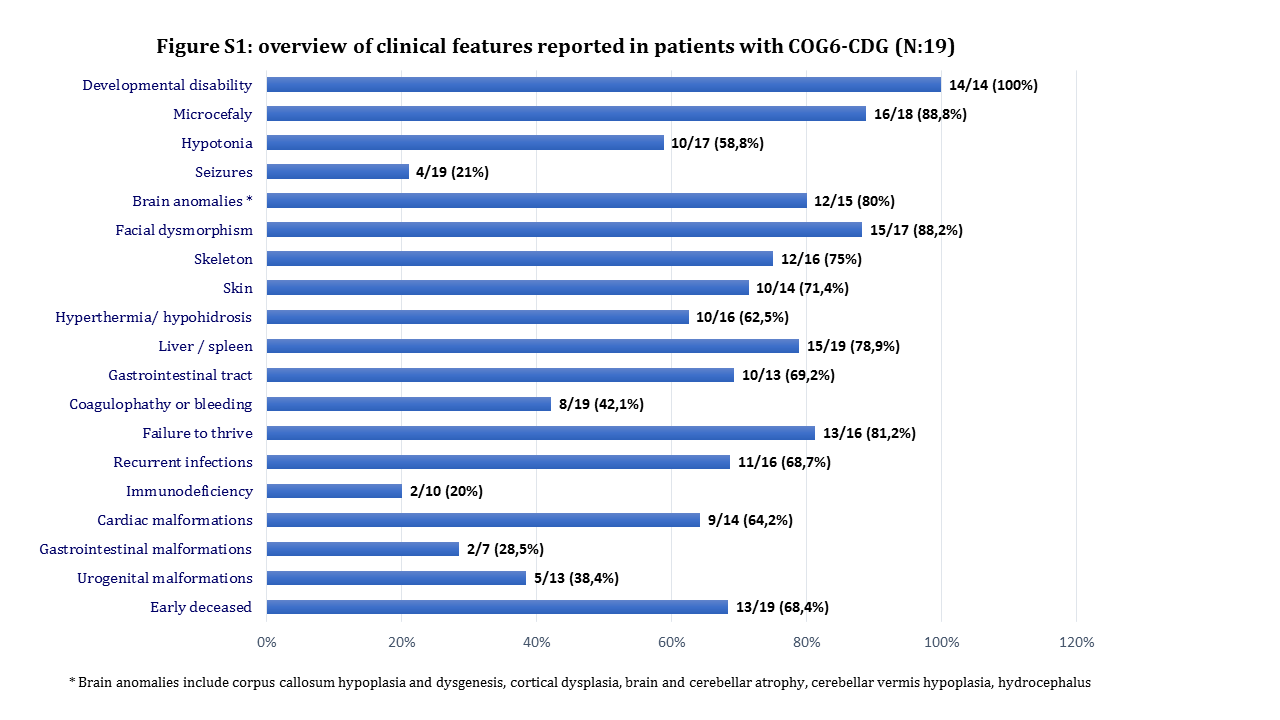

Supplement: Supplementary file 1 — Figure S1 Overview of clinical features reported in patients with COG6‐CDG (N:19) [file BDR2-114-165-s001.tif]
